# ASO Author Reflections: Pancreaticoduodenectomy for Isolated Para-Aortic Lymph Node Metastasis in Non-pancreatic Periampullary Cancers: Advocating for Careful Patient Selection and Routine Frozen Section

**DOI:** 10.1245/s10434-024-15957-8

**Published:** 2024-08-05

**Authors:** Vikram A. Chaudhari, Kaival Gundavda, Manish Bhandare, Shailesh V. Shrikhande

**Affiliations:** Division of Gastrointestinal and HPB Surgery, Department of Surgical Oncology, Tata Memorial Hospital, Homi Bhabha National Institute (HBNI), Mumbai, Maharashtra India

## PAST

Para-aortic lymph node (PALN) (Station 16b1) involvement is considered to represent metastatic disease in pancreatic and nonpancreatic periampullary cancers (NPPAC). Pancreaticoduodenectomy (PD) is inherently associated with significant morbidity, though mortality rates at high-volume centers have decreased to 2–3%.^1^ The potential survival benefit of radical resection in advanced or oligometastatic disease needs to justify the morbidity and potential mortality risk associated with surgery. Studies on the role of PALN dissection in isolated PALN involvement are mostly retrospective.^2,3^ They seem to suggest a benefit in patients who undergo radical resection and complete systemic therapy. NPPAC form more than 60% of our pancreatic resections.^1^ NPPAC are considered “prognostically better behaving” tumors compared with pancreatic ductal adenocarcinoma (PDAC). On this background, we sought to understand the role of PD in NPPAC with isolated PALN involvement, at our centre.^4^ We hypothesize that in patients with NPPAC and limited PALN involvement as the sole site of metastasis, radical resection may offer a potential survival benefit compared with chemotherapy alone.

## PRESENT

In this study,^5^ PD in patients isolated PALN metastasis was associated with survival comparable to regional node-positive patients [median overall survival (OS) 26.2 months versus 28.4 months, *p* = 0.33]. Additionally, patients who underwent resection had significantly better survival compared with those in whom resection was abandoned, in view of high surgical risk (comorbidities, high fistula risk score, advanced age, etc.) or extensive disease burden (26.2 months versus 9 months, *p* = 0.001). Furthermore, PALN dissection did not lead to increased morbidity, chyle leak rates, or mortality when compared with standard resections.

## FUTURE

Study findings suggest that PALN sampling and frozen section analysis of these nodes should be performed routinely during PD. It is unlikely to add any significant morbidity to the procedure, should improve nodal staging, and provides an opportunity to make an informed decision to either offer resection or abandon it in PALN-positive patients. Extensive nodal disease burden precluding technical R0 resection, advanced age, severe comorbidities, or patients with high fistula risks may serve as important criteria to defer surgery in this clinical situation. Future studies should focus on refining patient selection criteria and further optimizing treatment protocols for isolated PALN metastasis.
